# Spatial analysis of sexually transmitted infection vulnerability among pregnant women in Bandar Lampung: Policy implications for Indonesia’s Triple Elimination Program

**DOI:** 10.1016/j.ijregi.2025.100730

**Published:** 2025-08-19

**Authors:** Ratna Dewi Puspita Sari, Satriani Satriani, Dea Putri Andeska, Sutarto Sutarto

**Affiliations:** 1 Departement of Medical Education, Faculty of Medicine, Universitas Lampung, Lampung, Indonesia; 2 Departement of Environmental Science, Graduate School, Universitas Gadjah Mada, Mlati, Indonesia; 3 Departement of Biology, Faculty of Biology, Universitas Gadjah Mada, Mlati, Indonesia

**Keywords:** Geospatial vulnerability, Spatial epidemiology, Reproductive health, Infection screening, Maternal counseling

## Abstract

**Objectives:**

Sexually transmitted infections (STIs) remain a major public health concern, particularly among pregnant women, where vertical transmission poses severe risks to maternal and neonatal health. This study assessed the spatial distribution and vulnerability patterns of STIs among pregnant women in Bandar Lampung City, Indonesia, using a combination of geospatial and epidemiological analysis based on data collected in 2024.

**Methods:**

Data from 148 pregnant women screened through the national Triple Elimination Program were analyzed across 17 districts. A weighted vulnerability index was developed based on 10 sociodemographic and reproductive health factors, with Spearman’s correlation used to assess risk associations.

**Results:**

Spatial mapping revealed clustering of STI cases in central districts such as Enggal and Labuhan Ratu, whereas peripheral areas showed no recorded cases, possibly due to underreporting. Key vulnerability drivers included younger maternal age, multiparity, low education, and limited STI counseling. High hepatitis B virus prevalence (85.7% of cases) highlights persistent gaps in maternal STI prevention. District-level vulnerability mapping identified medium-risk zones requiring targeted intervention.

**Conclusions:**

Findings underscore the need for geographically focused screening, youth-friendly counseling, and integration of sexual health services into routine antenatal care to strengthen Indonesia’s Triple Elimination Program.

## Highlights

• Geospatial mapping of sexually transmitted infections in pregnant women in Bandar Lampung, Indonesia.
• Younger age, multiparity, low education, and limited counseling increased vulnerability.
• Hepatitis B virus dominated sexually transmitted infection cases, revealing prevention gaps in maternal health services.
• Medium-risk zones require geographically focused and youth-friendly interventions.
• Data support policy and triple elimination strategies in low-resource areas.

## Introduction

Sexually transmitted infections (STIs) remain a global health concern with serious consequences for reproductive, maternal, and neonatal health [[1], [2], [3]]. STIs refer to infections transmitted primarily through sexual contact, often without symptoms, whereas sexually transmitted diseases denote their clinical manifestations [4]. According to the World Health Organization, over one million STIs are acquired daily, many undiagnosed due to asymptomatic cases [5]. Each year, 374 million new infections are attributed to four curable STIs. Syphilis, in particular, poses grave risks during pregnancy, with around 930,000 cases annually causing approximately 350,000 adverse birth outcomes, including stillbirths, neonatal deaths, prematurity, and congenital disorders [6,7]. Hepatitis B virus (HBV) also represents a major health challenge in Indonesia, part of the Asia-Pacific region with a high burden of infection. Although national HBV prevalence declined from 9.4% in 2007 to 7.1% in 2013, the country is still considered moderately to highly endemic [8]. Vertical transmission during childbirth accounts for up to 95% of infections, posing serious risks such as chronic liver disease and cancer. As of 2019, 1.8% of pregnant women in Indonesia tested positive, with rates in some provinces exceeding 5% [[9], [10], [11]].

Indonesia introduced the HBV vaccination program for infants in 1997 and, by 2003, had scaled up nationwide delivery of the birth dose using prefilled single-use injection devices that remain effective outside the cold chain. This innovative strategy significantly improved vaccine coverage and contributed to a decline in chronic HBV infections across the general population [8]. Indonesia’s national guideline for the prevention of vertical transmission of STIs is outlined in the Ministry of Health Regulation No. 52 of 2017, which integrates routine antenatal screening for HIV, syphilis, and HBV into primary maternal care services. Building on this foundation, Indonesia formalized its approach to preventing mother-to-child transmission (MTCT) of STIs through the Ministry of Health Regulation No. 52 of 2017, which mandates routine antenatal screening for HIV, syphilis, and HBV in primary maternal care services. In 2018, the government launched the Triple Elimination Program, aiming to eliminate vertical transmission of all three infections [9]. Although the initiative marked important progress, significant implementation challenges remain [12]. The HBV component also struggles due to limited surveillance data. In early 2024, 6825 cases were clinically diagnosed and 18,985 confirmed in laboratories, with pregnant women making up the largest group affected (7056 cases) [13].

Although national efforts aim to improve screening and treatment, limited data on STI vulnerability patterns among pregnant women hinder targeted interventions. Disparities in health care access, socioeconomic status, and awareness create regional variation in STI risks [14,15]. Although spatial analysis has been used in infectious disease studies [16,17], its application to STI research in Indonesia remains limited. However, evidence suggests that spatial methods can reveal how STIs intersect with neighborhood-level factors like poverty and housing conditions, offering valuable insights for equity-driven interventions [18].

In Indonesia, addressing early-life health care inequalities is critical to reducing long-term health risks [19,20]. This is especially urgent in Lampung Province, where health care equity remains low, with a Public Health Development Index score of 54.5 and a service provision index of 35.3 [21]. Bandar Lampung City, a growing urban center in Southern Sumatra, faces persistent health disparities despite its urban status. This study explores spatial patterns of STI vulnerability among pregnant women in Bandar Lampung, examining how health access, poverty, and population density shape risk. By integrating spatial and epidemiological analysis, this research aims to inform more targeted, equity-driven interventions for improving maternal sexual health in Indonesia.

## Method

### Study design

This study aimed to assess fetomaternal STI vulnerability and spatial clustering among pregnant women in Bandar Lampung using geospatial and epidemiological methods. A descriptive-analytic approach combined a case-control design to identify key risk factors, with a cross-sectional design to map the distribution and clustering of STI cases. Spatial epidemiology tools were employed to identify high-risk areas and inform targeted public health strategies.

### Data collection

Data were collected from all 17 districts in Bandar Lampung between January and September 2024. The target population included pregnant women attending antenatal care (ANC) services at 31 operational Community Health Centers (CHCs), each covering 5-9 urban villages. From medical records, pregnant women who had completed triple elimination screening for HIV, syphilis, and hepatitis B were identified. Based on this review, 148 women met the eligibility criteria and had complete screening records.

These women were subsequently visited at their residences by trained enumerators to verify medical record accuracy, collect GPS coordinates, and conduct structured interviews on sociodemographic and behavioral risk factors relevant to STI vulnerability. This multistage process ensured data completeness and spatial validity.

**Inclusion criteria** were (i) pregnant women receiving ANC services at CHCs in Bandar Lampung between January and September 2024, (ii) complete triple elimination screening results recorded in the health system, (iii) residence within the city’s administrative boundaries, and (iv) provision of informed consent.

**Exclusion criteria** included (i) incomplete screening records, (ii) inability to be reached or interviewed during follow-up, (iii) refusal to participate, (iv) missing or invalid GPS location data, and (v) medical conditions that precluded participation.

### Data analysis

A multidimensional vulnerability analysis was conducted using 10 indicators: age, occupation, education, Water, Sanitation, and Hygiene (WASH) access, STI counseling, parity, ANC attendance, sexual partner, contraceptive use, and prior STI during pregnancy. Each was scored on a three-point scale (low = 1, medium = 2, high = 3), based on district-level thresholds drawn from relevant literature and contextual insights (for more details, please refer to Supplementary Table 1).

The weighted vulnerability index (WVI) was constructed from these variables to quantify STI-related vulnerability at the respondent level. Using K-means clustering, districts were then categorized into low, medium, or high vulnerability zones. Spatial patterns of STI cases and WVI classifications were visualized using Quantum Geographic Information System (QGIS). The spatial analysis was conducted across 17 districts, which represent the complete set of administrative divisions in Bandar Lampung City. These units were selected intentionally to align with how Indonesia’s Triple Elimination initiative is implemented and targeted at the local level.

## Results

### Distribution of STIs in pregnant women in Bandar Lampung

Out of the total 148 respondents, 14 pregnant women were identified as having STIs based on the triple elimination screening results. Geographically, out of the 17 districts in Bandar Lampung City, eight districts reported at least one STI case, whereas the remaining nine recorded no cases during the study period.

**Figure 1:**
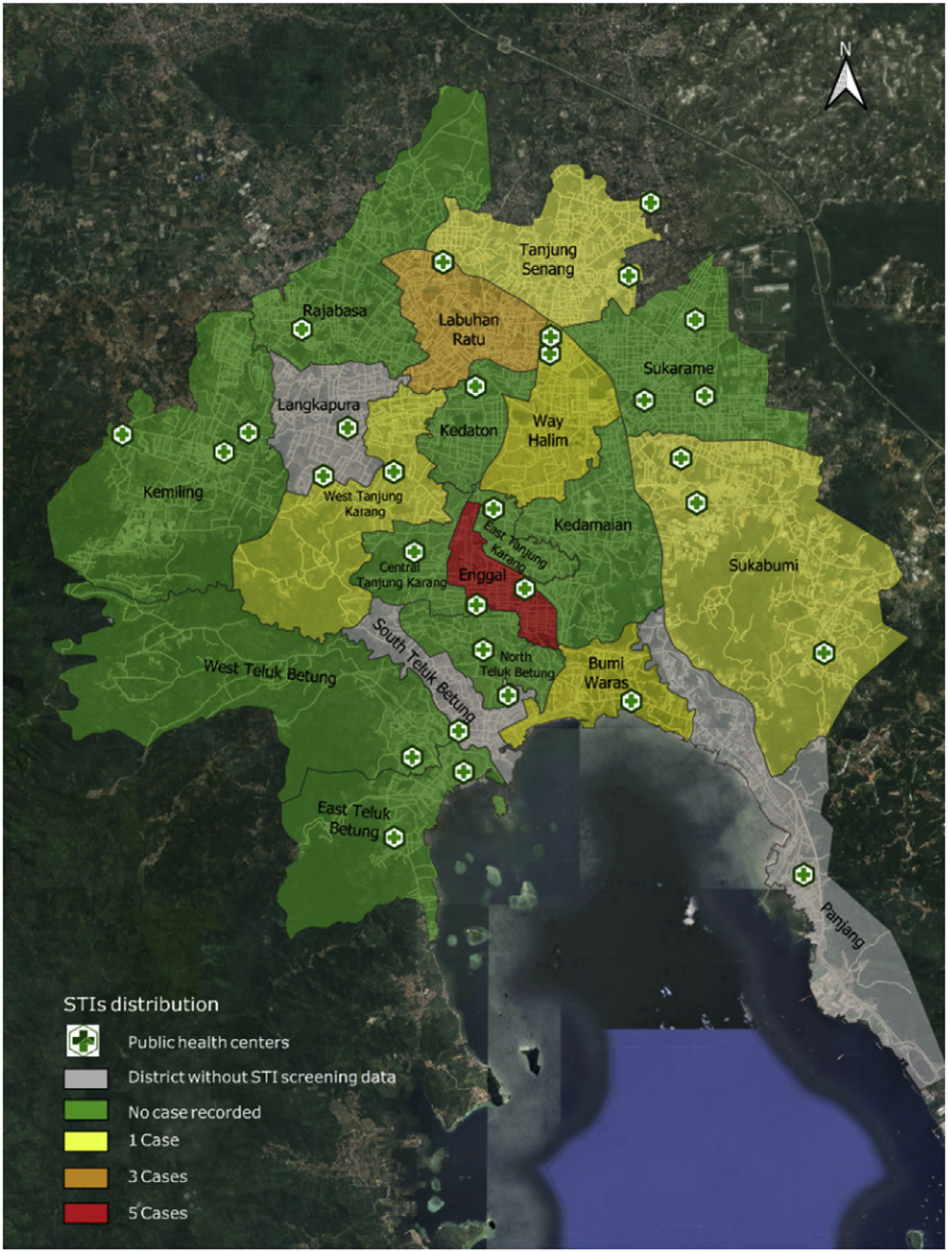
Spatial distribution of STIs among pregnant women in Bandar Lampung City. STI, sexually transmitted infection. This map illustrates the spatial distribution of STI cases among pregnant women across subdistricts in Bandar Lampung City, Indonesia. Colored zones represent varying case intensities: green for no cases, yellow for 1, orange for 3, and red for 5 cases. Grey areas denote districts lacking STI screening data. Locations of public health centers are also marked to visualize access to health services. Note: The pattern shown is based on visual mapping and does not reflect statistically tested clustering (Supplementary Figure 1).

HBV emerged as the most prevalent infection, accounting for 85.7% of total cases (12 out of 14). Although HBV can be transmitted both sexually and vertically, the exact route of transmission could not be determined in this study. In contrast, HIV and syphilis were each detected in only one case, both of which were found exclusively in Enggal District. This makes Enggal not only the district with the highest number of cases (n = 5), but also the only district with all three types of infections: HBV, HIV, and syphilis. Following Enggal, Labuhan Ratu recorded the second-highest number of cases (n = 3), all of which were HBV. Five other districts (i.e., Bumi Waras, Kedaton, Sukabumi, Tanjung Senang, Tanjung Karang Barat, and Way Halim) each reported a single case of HBV (Supplementary Figure 1). The spatial distribution of STI cases is visualized in Figure 1, highlighting a concentration of cases in central districts such as Enggal and Labuhan Ratu. This visual pattern suggests potential spatial clustering; however, no statistical test for spatial autocorrelation was performed.

### Key risk factors contributing to STIs

The analysis identified 10 key risk factors contributing to STI vulnerability using Spearman’s rank correlation among pregnant women in Bandar Lampung. The detailed results are shown in Table 1, Table 2. The correlation results are presented in Table 3. The factor most strongly associated with STI risk was the number of sexual partners (ρ = 0.610, *P* = 0.0000), followed by STI counseling (ρ = 0.447, *P* = 0.0000), WASH access (ρ = 0.296, *P* = 0.0003), parity (ρ = 0.279, *P* = 0.0006), and prior infection during pregnancy (ρ = 0.230, *P* = 0.005). Protective associations were observed for maternal age (ρ = -0.292, *P* = 0.0003), ANC attendance (ρ = -0.182, *P* = 0.027), and education level (ρ = -0.196, *P* = 0.017). Occupation and contraceptive use showed no statistically significant relationship with STI risk (*P* >0.05).

**Table 1:** Spearman correlation matrix of STI risk factors.

ANC, antenatal care; STI, sexually transmitted infection.

This matrix presents the pairwise Spearman correlation coefficients (ρ) between total STI cases and various demographic, behavioral, and service-related factors. Strong positive or negative correlations are highlighted to indicate significant relationships between variables.

**Table 2:** Spearman correlation coefficients and statistical significance of STI risk factors.

Factor	Spearman rho (ρ)	*P*-value	Interpretation
Age	–0.292	0.0003	Older women have a lower risk of STIs (significant)
Occupation	–0.083	0.3177	No significant association with STI risk
Education	–0.196	0.0168	Higher education is associated with lower STI risk
STI counseling	**0.447**	**0.0000**	Attending counseling is linked with higher STI cases (likely reverse causality)
WASH access	0.296	0.0003	Better WASH access is associated with higher STI risk (urban exposure factor)
Parity	0.279	0.0006	More childbirths increase STI vulnerability
ANC attendance	–0.182	0.0270	More ANC visits reduce STI risk (protective factor)
Sexual partner	**0.61**	**0.0000**	More sexual partners strongly increase STI risk
Contraception use	–0.034	0.6808	No significant relationship with STI risk
Infection during pregnancy	0.23	0.0050	Previous infections during pregnancy increase vulnerability

ANC, antenatal care; STI, sexually transmitted infection.

This table summarizes the Spearman rho (ρ) values and corresponding *P*-values for the association between selected factors and STI incidence among pregnant women. Statistically significant associations (*P* <0.05) are interpreted in terms of their potential contribution to STI vulnerability or protection.

**Table 3:** Spatially informed recommendations for reducing STI vulnerability among pregnant women in Bandar Lampung.

Priority area	Target districts	Recommended actions
Routine STI screening	Medium-risk zones	Expand testing for syphilis, HIV, and hepatitis B virus during ANC include partner testing.
Youth-friendly counseling	All districts, focus on areas with younger maternal population (e.g., Kedaton, Enggal)	Offer STI prevention for pregnant women aged 15-24 and male partners.
Multiparous women support	Medium-risk zones and urban centers	Integrate family planning and repeat screening into ANC.
Health worker training	City-wide	Train providers for preventive, not just reactive, STI counseling.
Urban risks mitigation	Medium-risk urban districts (e.g., Bumi Waras, Teluk Betung)	Combine WASH improvements with behavior change and education.
Data surveillance strengthening	Data-deficient districts (e.g., Langkapura, Panjang)	Expand routine STI reporting and baseline screening.

ANC, antenatal care; STI, sexually transmitted infection.

### Categorization of STI vulnerability through spatial and cluster analysis

The WVI was constructed by aggregating multiple standardized indicators, as outlined in Supplementary Table 1. The resulting vulnerability map (Figure 1**)** revealed a clear spatial stratification of STI risk across districts. Specifically, districts such as Rajabasa, Tanjung Senang, and Sukarame, located in the northern region, were classified as low-risk zones, as denoted by the green shading. These districts consistently scored one across most indicators, especially on critical factors such as education, ANC, and number of sexual partners. Although some districts exhibited high vulnerability in one or two indicators, the generally low scores across other variables appeared sufficient to maintain an overall low vulnerability level. For instance, in Rajabasa District, four indicators (i.e., occupation, parity, contraception use, and infection during pregnancy) recorded relatively high scores of 3, 2, 3, and 3, respectively. However, the remaining six indicators, including age, education, STI counseling, access to WASH, ANC, and number of sexual partners, consistently scored low at level 1 (for more details, please refer to Supplementary Table 2).

**Figure 2:**
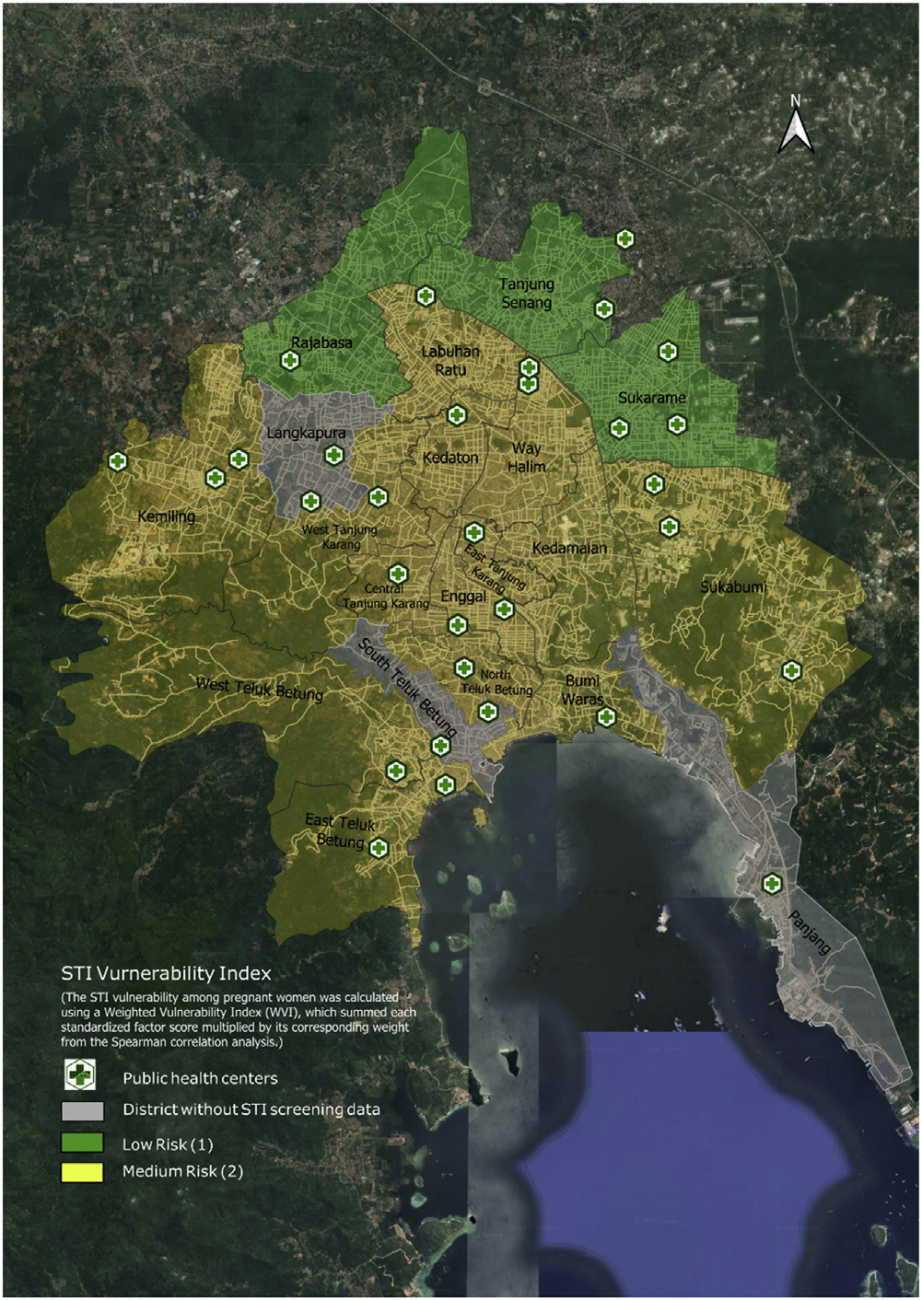
Spatial distribution of STIs vulnerability index among pregnant women in Bandar Lampung City. STI, sexually transmitted infection. The STI vulnerability index was calculated using a weighted standardized index method, based on selected risk factors with significant Spearman correlation values. The map illustrates districts categorized into low and medium vulnerability levels. Public health center locations and districts without STI screening data are also indicated.

In contrast, districts highlighted in yellow were classified as medium risk according to the WVI. Despite a relatively even distribution of public health centers across the region, this did not necessarily correspond with reduced STI vulnerability, particularly within these medium-risk districts. Moreover, several districts, including Langkapura, South Teluk Betung, and Panjang, lacked STI screening data, as indicated by the gray shading on the map (Figure 2).

## Discussion

### Understanding STI risk through spatial patterns and cluster analysis

The low detection of reported STI cases across Bandar Lampung’s districts likely reflects limitations in screening coverage and surveillance rather than a true absence of infection. Many STIs are asymptomatic or latent, resulting in underdiagnosis without proactive screening [22]. Structural and systemic barriers, including social stigma, inadequate health care provider training, and poor integration of diagnostic services, further contribute to underreporting and influence the observed STI distribution among pregnant women [22,23].

The high prevalence of STIs, particularly HBV, aligns with global and national recognition of HBV as a major public health concern, especially in low- and middle-income countries [24]. Although HBV is included in this analysis due to its inclusion in the Triple Elimination Program, we acknowledge that not all cases in pregnant women are necessarily sexually transmitted. However, the strong statistical association between sexual behavior (e.g., multiple partners) and infection risk suggests that sexual transmission remains a significant contributor to maternal vulnerability. Therefore, inclusion of HBV under the broader STI vulnerability framework is programmatically and analytically relevant.

District-level variations may stem from human mobility, which facilitates transmission and leads to higher detection in areas with better health care access [16,17]. However, the presence of CHCs does not guarantee higher case detection, highlighting the role of awareness, stigma, and screening uptake [25,26]. For example, Enggal, a densely populated district, exemplifies how increased exposure and social complexity can heighten STI risk despite improved service availability. This uneven distribution underscores the need for geographically targeted interventions, particularly in districts with limited health care accessibility or insufficient surveillance data [27,28]. The notably high HBV prevalence among pregnant women calls for integrating STI prevention into maternal health services, especially in areas with weak monitoring systems, to prevent hidden transmission. Strengthening local health data systems and capacity building among health care workers are essential to the success of such efforts.

Spatial analysis revealed that certain districts, such as Rajabasa, remain categorized as low-risk zones because protective factors offset elevated risk factors. In high-vulnerability areas, poor reproductive health awareness emerges as a major obstacle to preventive understanding and action. High multiparity and low utilization of STI counseling indicate informational and access barriers, particularly among younger women with limited health literacy [29]. These conditions increase risk by undermining decision-making capacity and care-seeking behavior.

These patterns underscore the importance of geographically targeted interventions, especially in medium and high-vulnerability areas. Crucially, the presence of facilities alone is insufficient if services fail to address behavioral and social dimensions of health care utilization [4,22]. This gap represents a blind spot within surveillance and service delivery systems that could weaken Triple Elimination Program efforts targeting HIV, syphilis, and HBV [30]. The generated vulnerability maps underscore the importance of geographically targeted interventions focusing on moderate-risk districts and expanding screening coverage in areas with incomplete data. Evidence from Ethiopia and Guangzhou supports spatial analysis as a valuable tool for identifying risk clusters and guiding targeted responses [31].

### Interlinked drivers of STI vulnerability: reproductive and sociodemographic dimensions

Behavioral and sociodemographic factors jointly shape STI vulnerability among pregnant women. Behavioral factor is a key contributor to STI vulnerability among pregnant women. Globally, having multiple sexual partners and inconsistent condom use remain major risk factors [14,32]. The role of STI counseling warrants particular attention. Although intended as a preventive measure, the data suggest potential reverse causality, wherein symptomatic or high-risk women are more likely to seek counseling [32]. This highlights missed opportunities for early prevention and underscores the need for proactive, empowerment-focused counseling models to enhance STI prevention effectiveness.

Multiparity increases STI risk by compounding exposure and reflecting barriers to preventive services, especially for infections like syphilis [33]. In addition, complications during pregnancy may signal underlying conditions that facilitate STI transmission and progression. ANC services, while offering vital opportunities for screening and education, showed only moderate protective effects, likely due to uneven integration of sexual health components [34]. Attendance tends to increase among multiparous women with prior complications, underscoring how reproductive history influences care-seeking. Meanwhile, contraceptive use was not protective, likely due to widespread use of non-barrier methods. This underlines the need to promote condom use in family planning efforts [32,35].

Among sociodemographic variables, age was the most significant risk factor. This finding reinforces the necessity for age-tailored interventions that not only reduce risk behaviors but also address structural barriers to ensure youth-friendly STI prevention services [36]. Younger populations are less likely to utilize health care services effectively and achieve viral suppression in the case of HIV, perpetuating transmission cycles and elevating vulnerability [37].

Education serves as a significant protective factor by enhancing health literacy, enabling women to access, comprehend, and apply sexual and reproductive health information more effectively [38]. The well-established relationship between female education and improved health outcomes highlights educational investment as both a social goal and a long-term strategy to curb STI prevalence [39]. An unexpected positive association emerged between access to WASH and STI risk. This paradox may reflect the urban environment where improved infrastructure coincides with behaviors increasing sexual risk. Integrating WASH initiatives with sexual and maternal health services could offer synergistic benefits, advancing both STI prevention and broader health goals under the Sustainable Development Goals (SDGs).

### Implications for the triple elimination program

The spatial and statistical findings from this study offer critical implications for Indonesia’s Triple Elimination Program, which targets the eradication of MTCT of HIV, syphilis, and HBV. The STI Vulnerability Index map reveals clear spatial disparities. Districts in the southern and central parts of Bandar Lampung, including Bumi Waras, Enggal, Kedamaian, and Teluk Betung areas, emerged as medium-risk zones. These areas overlap with urban settlements where previous analysis identified higher rates of multiparity, limited STI counseling coverage, and younger maternal age, factors that compound vulnerability. In contrast, northern districts such as Tanjung Senang, Rajabasa, and Sukarame are categorized as low-risk, aligning with better ANC attendance and higher education levels among women. This clustering highlights the need for geographically targeted interventions. Prioritizing screening, health promotion, and partner treatment in medium-risk areas can accelerate progress. For instance, scaling up routine screening for syphilis, HIV, and HBV during ANC visits, already part of national guidelines, can help prevent vertical transmission, particularly in districts such as Bumi Waras and Enggal, where vulnerability is the highest.

Meanwhile, gray-shaded districts on the map indicate missing screening data, underscoring surveillance gaps that risk leaving vulnerable areas underserved. Interventions should reflect spatial and demographic risks [16,18], integrating STI services into maternal care, enhancing surveillance, and addressing barriers such as stigma, limited tools, and fragmented systems [11,32]. District-specific strategies, like youth-focused counseling, multiparity support, and proactive health worker training, can drive progress toward Triple Elimination goals.

In summary, the findings call for a multifaceted, spatially responsive strategy: scaling up routine screening, tailoring services for high-risk groups (younger, multiparous, and less-educated women), addressing urban-specific risks, and closing surveillance gaps in unscreened districts. Aligning service delivery with geographic and demographic vulnerability can accelerate progress toward eliminating MTCT of STIs. However, key challenges remain, including data gaps, underreporting, and sociocultural barriers that hinder detection and care-seeking. Future research should incorporate qualitative insights on partner dynamics, stigma, and service quality, while applying predictive geospatial models to strengthen targeting and early warning. A stronger evidence base and more precise interventions will ensure that Indonesia’s STI prevention efforts remain adaptive, inclusive, and aligned with broader maternal and child health goals.

## Conclusion

This study reveals significant spatial and demographic disparities in STI vulnerability among pregnant women in Bandar Lampung. STI cases cluster in central urban districts, with elevated risk driven by younger maternal age, multiparity, low education, and limited STI counseling. Despite widespread health facility availability, service uptake and quality remain uneven, especially in medium-risk areas. A spatially targeted response is needed, focusing on youth-oriented counseling, integrated antenatal screening, and partner engagement. Strengthening surveillance and addressing data gaps in unscreened districts are also vital for equitable coverage. Aligning STI prevention with local vulnerability patterns can enhance the effectiveness and inclusivity of Indonesia’s Triple Elimination initiative.

### Limitations

This study has several limitations. The vulnerability index was developed from a limited number of pregnant women who had complete triple elimination screening and valid spatial data. Although the data were drawn from all eligible cases across 31 public health centers, the findings may not fully represent populations outside the public health system. The use of 17 administrative districts may also limit the statistical power of spatial analyses, although these units reflect the operational structure of local health programs. In addition, the cross-sectional design limits causal inference, and some risk factors, such as partner networks or mobility, were not captured. Despite these limitations, the study benefits from verified clinical records, spatial mapping, and structured follow-up, offering valuable insights into maternal STI vulnerability in an urban Indonesian setting.

## Declaration of competing interest

The authors have no competing of interests to declare.

## Appendix Supplementary materials

Image, application 1

Image, application 2

Image, application 3
