# Supplementary material for: Spatial analysis of sexually transmitted infection vulnerability among pregnant women in Bandar Lampung: Policy implications for Indonesia’s Triple Elimination Program
Source: IJID Reg. 2025 Aug 19;16:100730. doi: 10.1016/j.ijregi.2025.100730 (PMC12445606; doi:10.1016/j.ijregi.2025.100730)
Supplement: Supplementary file 1 [file mmc1.docx]

**Fig. 1.** Distribution of STIs among Pregnant Women by District in Bandar Lampung City

This bar chart shows the distribution of HBV, syphilis, and HIV cases among pregnant women across districts in Bandar Lampung. HBV was the most common infection, concentrated in Enggal and Labuhan Ratu, while syphilis and HIV appeared only in Kedamaian District.
