# Supplementary material for: Spatial analysis of sexually transmitted infection vulnerability among pregnant women in Bandar Lampung: Policy implications for Indonesia’s Triple Elimination Program
Source: IJID Reg. 2025 Aug 19;16:100730. doi: 10.1016/j.ijregi.2025.100730 (PMC12445606; doi:10.1016/j.ijregi.2025.100730)
Supplement: Supplementary file 2 [file mmc2.docx]

**Table 1.** Scoring Criteria for STI Vulnerability Factors

| **Factor** | | **Vulnerable Subgroup Description** | **Low**  **(1)** | **Medium**  **(2)** | **High (3)** |
| --- | --- | --- | --- | --- | --- |
| **Socio-demographic factors** | Age | % of pregnant women aged 15–24 years | <10% | 11% – 20% | ≥ 21% |
|  | Occupation | % of pregnant women who are unemployed | < 55% | 56% – 78% | ≥79% |
|  | Education | % of pregnant women with ≤12 years of education | <33% | 31% – 67% | ≥68% |
|  | WASH Access | % of pregnant women with no access to clean water and sanitation | <47% | 48% – 73% | ≥74% |
| **Reproductive health factors** | STI Counseling | % of pregnant women who did not receive STI counseling during ANC visit | < 30% | 31% – 60% | >61% |
|  | Parity | % of pregnant women who have multiparous | <33% | 34% – 67% | ≥68% |
|  | ANC Attendance | % of pregnant women with <4 ANC visits during pregnancy | <11% | 12% – 22% | ≥23% |
|  | Sexual Partners | % of pregnant women who reported multiple sexual partners | <7% | 8% – 13% | ≥14% |
|  | Contraception Use | % of pregnant women who did not use condoms as contraception | <33% | 34% – 67% | ≥68% |
|  | Infection During Pregnancy | % of pregnant women who experienced any infection during pregnancy | <11% | 12% – 22% | ≥23% |

Spearman’s rank correlation was used to measure the association between each factor and the total number of STI cases per district. The remaining factors were assigned weights proportional to their correlation coefficients, allowing for the construction of a Weighted Vulnerability Index (WVI) for each district. The WVI was calculated as the sum of each standardized factor score multiplied by its corresponding weight.
