# Supplementary material for: Spatial analysis of sexually transmitted infection vulnerability among pregnant women in Bandar Lampung: Policy implications for Indonesia’s Triple Elimination Program
Source: IJID Reg. 2025 Aug 19;16:100730. doi: 10.1016/j.ijregi.2025.100730 (PMC12445606; doi:10.1016/j.ijregi.2025.100730)
Supplement: Supplementary file 3 [file mmc3.docx]

**Tabel 2.** Summary of STIs Vulnerability Factor

| **District** | **Age** | **Ocupation** | **Education** | **STI Counseling** | **WASH Acces** | **Parity** | **ANC** | **Sexual Partnert** | **Contraception Use** | **Infection during Prenancy** | **STI Vulnerability Index (WVI)** |
| --- | --- | --- | --- | --- | --- | --- | --- | --- | --- | --- | --- |
| Bumi Waras | 2 | 3 | 1 | 3 | 2 | 3 | 3 | 2 | 3 | 1 | **2** |
| Enggal | 1 | 3 | 1 | 3 | 3 | 3 | 1 | 3 | 3 | 2 | **2** |
| Kedamaian | 3 | 3 | 3 | 1 | 3 | 1 | 1 | 1 | 3 | 1 | **2** |
| Kedaton | 1 | 3 | 3 | 1 | 3 | 3 | 1 | 1 | 1 | 1 | **1** |
| Kemiling | 2 | 3 | 2 | 2 | 1 | 3 | 1 | 3 | 3 | 1 | **2** |
| Labuhan Ratu | 2 | 1 | 1 | 2 | 2 | 3 | 1 | 3 | 3 | 1 | **2** |
| Rajabasa | 1 | 3 | 1 | 1 | 1 | 2 | 1 | 1 | 3 | 3 | **1** |
| Sukabumi | 1 | 2 | 1 | 2 | 2 | 2 | 1 | 1 | 3 | 2 | **1** |
| Sukarame | 1 | 1 | 1 | 1 | 3 | 2 | 1 | 1 | 3 | 1 | **1** |
| Tanjung Karang Barat | 2 | 3 | 1 | 2 | 1 | 3 | 2 | 1 | 3 | 1 | **1** |
| Tanjung Karang Pusat | 2 | 3 | 1 | 2 | 2 | 3 | 2 | 1 | 3 | 1 | **2** |
| Tanjung Karang Timur | 2 | 3 | 1 | 2 | 3 | 3 | 1 | 2 | 3 | 1 | **2** |
| Tanjung Senang | 2 | 2 | 1 | 2 | 2 | 1 | 1 | 1 | 3 | 1 | **1** |
| Teluk Betung Barat | 3 | 3 | 2 | 2 | 1 | 3 | 2 | 1 | 3 | 1 | **2** |
| Teluk Betung Timur | 3 | 3 | 1 | 1 | 1 | 2 | 3 | 1 | 3 | 1 | **1** |
| Teluk Betung Utara | 1 | 1 | 1 | 3 | 2 | 2 | 1 | 1 | 3 | 1 | **2** |
| Way Halim | 2 | 3 | 1 | 2 | 2 | 2 | 1 | 1 | 3 | 2 | **2** |
